# Has incentive payment improved venous thrombo-embolism risk assessment and treatment of hospital in-patients?

**DOI:** 10.12688/f1000research.2-41.v1

**Published:** 2013-02-12

**Authors:** Sue Child, Rod Sheaff, Olga Boiko, Alice Bateman, Christian A Gericke

**Affiliations:** 1 Peninsula CLAHRC, National Institute for Health Research, The University of Exeter Medical School, Plymouth, PL4 8AA, UK; 2 Peninsula CLAHRC, National Institute for Health Research, Peninsula College of Medicine and Dentistry, Plymouth University, Plymouth, PL4 8AA, UK; 3 Primary Care Research Team, The University of Exeter Medical School, Exeter, EX1 2LU, UK; 4 The Wesley Research Institute, Brisbane, QLD 4066, Australia

## Abstract

This paper focuses on financial incentives rewarding successful implementation of guidelines in the UK National Health Service (NHS). In particular, it assesses the implementation of National Institute for Health and Clinical Excellence (NICE) venous thrombo-embolism (VTE) guidance in 2010 on the risk assessment and secondary prevention of VTE in hospital in-patients and the financial incentives driving successful implementation introduced by the Commissioning for Quality and Innovation for Payment Framework (CQUIN) for 2010-2011. We systematically compared the implementation of evidence-based national guidance on VTE prevention across two specialities (general medicine and orthopaedics) in four hospital sites in the greater South West of England by auditing and evaluating VTE prevention activity for 2009 (i.e. before the 2010 NICE guideline) and late 2010 (almost a year after the guideline was published). Analysis of VTE prevention activity reported in 816 randomly selected orthopaedic and general medical in-patient medical records was complemented by a qualitative study into the practical responses to revised national guidance. This paper’s contribution to knowledge is to suggest that by financially rewarding the implementation of national guidance on VTE prevention, paradoxes and contradictions have become apparent between the ‘payment by volume system’ of Healthcare Resource Groups and the ‘payment by results’ system of CQUIN.

## Background to incentive payments in the NHS

Diagnostic Related Groups (DRGs) offered health organisations the ability to understand provider activity in terms of how many patients they cared for and their case-mix. They allowed activity comparison within and between different organisations as well as providing a means of making longitudinal analyses of trends in treatment and service provision. Local adaptations or variants of diagnosis related groups (DRGs) have been adopted as the unit of payments to hospitals in many health systems ^1^. This is a system that classifies hospital cases into identifiable groups in order to identify ‘products’ that a hospital provides. The system was originally developed in the USA in order to replace ‘cost-based’ reimbursement. DRGs are based on the International Classification of Diseases (ICD) and the presence of complications or co-morbidity. This model of classification has spread to other countries including Australia, Denmark, France, Germany, Austria, the Netherlands and Russia; followed by a less rapid spread from acute care into long-term care and psychiatry ^2^.

In England, the NHS Plan of July 2000 introduced the government’s intention to link the allocation of hospital funds to the activity they undertake – a system of payment by results (PbR) ^3^. PbR underpinned the NHS reform agenda and was formally implemented by The Department of Health in 2002 as a mechanism to reimburse hospitals in England for levels of activity undertaken. Under this new system of PbR hospitals were reimbursed for activities undertaken using a fixed price tariff that reflected national average prices for hospital procedures ^4^. Such prices are standardised across the NHS with adjustments for higher labour costs in London. The main aim of PbR was to improve efficiency and increase value for money in the NHS by enhancing service quality, facilitating patient choice, enabling service innovation, improving quality of service and reducing waiting times by rewarding healthcare providers for the volumes of work completed.

PbR has continued to be implemented in the NHS (albeit incrementally) both in terms of scope and financial impact. The first NHS foundation trust (FT) applicants moved to the full PbR system in 2005–06 and other NHS trusts in 2006–07 ^4^. In 2006–2007 PbR was extended to include non-elective, outpatient and emergency admissions for all trusts ^5^. In 2009–2010, the national tariff for admitted patient care and outpatient activity was for the first time defined in terms of Healthcare Resource Groups (HRGs), an English variant of DRGs. The Department of Health in the UK defines HRGs as groupings of treatment episodes that are similar in resource use and clinical response. They are a standard method of analysing clinical procedures and thereby classification of hospital activity ^6^ HRGs are reviewed every four years to ensure they accurately reflect clinical practice.

Although the imposition of payment by results was intended as a fair and consistent basis for hospital funding it also rewards hospitals by volume of work completed. Its overarching aim was to allow the commissioning of activity volumes that were required to deliver service priorities from a plurality of providers on the basis of a standard, national pricing tariff. By standardising prices, it was intended to promote non-price competition, i.e. provider differentiation on the basis of service quality ^4^.

NHS commissioners therefore faced a very challenging agenda. They needed to secure high quality services which remained safe for patients, whilst at the same time trying to achieve best value for the budgets available.

## The CQUIN (Commissioning for Quality and Innovation) framework

Further policy aims were in play however. Commissioning specifically to improve clinical quality and safety required additional incentive frameworks through which improvements could be monitored and additional drivers for change generated. Alongside the more traditional payment by results (volume), there was apparently a need to develop more effective patient experience metrics and use these to improve quality and safety. Lord Darzi’s review of the NHS: "High Quality Care for All" ^7^ included a commitment to make providers’ income linked to quality of care performance. This commitment led to the creation of the Commissioning for Quality and Innovation (CQUIN) payment framework, introduced nationally in 2009 to help local commissioners identify and agree quality improvement schemes. It enabled commissioners to reward excellence by linking a proportion of healthcare providers’ income to the achievement of local quality improvement goals ^8^. CQUIN targets introduced for VTE in 2010–11 included a financial incentive for reaching a 90% threshold for venous thrombo-embolism (VTE) risk assessment of all adult hospital in-patients. Compliance was to be measured through a monthly audit of patients who received appropriate thrombo-prophylaxis after risk assessment.

### Why did CQUIN focus on VTE?

It has been over 23 years since the first evidence-based guidelines ^9^ recommended routine use of thromboprophylaxis for most hospitalised patients but there continue to be large gaps in the provision of this key patient safety intervention. Venous thromboembolism continues to be a major and often unrecognised cause of morbidity and mortality in hospitalised patients ^10^. Over one-third of hospital in-patients are at risk of developing venous thrombo-embolism (VTE) ^11^. This is a condition where a blood clot forms in a vein and travels through the bloodstream to the lungs, where it often proves fatal.

More recently, national efforts to reduce avoidable deaths from VTE recommenced in 2005, when the House of Commons Health Select Committee estimated that over 25,000 patients admitted to hospital died each year from preventable VTE ^11^. The Committee cited evidence that many deaths might had been avoided if patients had been properly assessed for risk of VTE on admission to hospital and received appropriate prophylaxis ^12^. They concluded that a nationwide system was needed in the NHS to identify patients at risk of VTE on admission to hospital as well as taking steps to ensure that appropriate thrombo-prophylaxis was prescribed unless contra-indicated.

As a result a financial incentive for hospitals to focus on VTE risk assessment and prevention was introduced through the Commissioning for Quality and Innovation for Payment Framework (CQUIN) for 2010–2011 ^13^. A percentage of CQUIN payments paid to or withheld from acute providers became conditional on risk assessing for VTE at least 90% of patients admitted to hospital as in-patients. As well as acting as a monitor for ensuring delivery of risk assessment procedures, this measure also supported VTE prevention by providing a pan-NHS picture of compliance.

There are also national requirements for all acute hospitals to undertake and report a monthly audit of the percentage of their patients who have received appropriate thrombo-prophylaxis after risk assessment and undertake a root cause analysis of confirmed cases of hospital acquired VTE. Further audit requirements include the reassessment of risk after 24 hours and the provision of patient information on VTE prevention. Compliance with all these indicators is measured from a nationally mandated monthly Unify2 data collection for all providers of NHS acute services. Failure to report in line with such contractual obligations allows commissioners to withhold a percentage of contract value.

## Paradoxes and contradictions within the CQUIN payment framework

What is interesting about the CQUIN payment framework is that it enables commissioners to reward excellence by linking a proportion of English healthcare providers’ income to the achievement of local quality improvement goals. Unlike incentive systems that reward *individuals*, CQUIN rewards are made available to *providers* through contracts with primary care trusts (PCTs). CQUIN funding is up to 1.5% of contract outturn value, conditional upon achievement of quality outcomes specified in the contract. The contract outturn value is a locally agreed package of quality improvement goals and indicators, which, if achieved enables the provider to earn its full CQUIN payment (calculated as 1.5% of the actual outturn value of the provider contract). The financial value of a CQUIN scheme is specified nationally as a % of actual outturn value of the provider’s contract. At the start of the year, the expected value of the scheme should be calculated as a % of the estimated total outturn contract value for commissioned patient services. A year-end reconciliation is then required against actual outturn value ^13^.

Therefore, it might be questioned what motivates individuals working for PCTs to successfully implement local quality improvement goals when they have no *personal* benefit in terms of monetary reward. The following section discusses different types of motivation and how each may act as either a barrier or facilitator to the successful implementation of such ‘payment by results’ schemes in the wider NHS.

### Motivation in the NHS

In virtually every developed country the health service is the largest single employer, and Britain is no exception. The desire to increase capacity within the NHS means that it has probably never been more important to understand what attracts, retains and motivates staff ^14^. The ninth annual survey to collect the views of NHS staff across England was published in 2011. It indicated that 90% of staff felt their role ultimately made a difference to patients, and 87% were satisfied with the quality of care they personally gave ^15^.

Yet, despite the individual (subjective) needs of each patient it may be argued that standardisation of effective clinical practice is becoming more commonplace. Risk assessing for VTE is one example. It would prove extremely difficult for compliance to be rewarded unless a standardised, objective system of directly comparable output existed. Revised NICE guidance in January 2010 formalised differing hospital guidelines for attributing VTE risk, administering appropriate prophylaxis and dealing with contraindications. This meant that compliance against a national target was now able to be monitored for every acute provider. As a result, directly measurable financial incentives for acute providers to focus on VTE risk assessment and prevention through the CQUIN payments framework were able to be introduced on a nationwide basis.

It would appear to be a rational assumption that offering any form of financial incentivisation should drive up employee compliance with new or revised working practices. An employee’s motivation to perform effectively is said to be determined by two variables ^16^. The first is contained in the effort-reward probability. This is determined by two further probabilities:

1. The probability that effort will result in performance.

2. The probability that performance will result in reward.

Yet, this would appear to be paradoxical to the aims of CQUIN. Here we see quite clearly that an increased effort by individuals to properly risk assess and treat VTE in hospital in-patients is not financially rewarding to themselves, but to the *provider* for whom they work.

What the CQUIN framework appears to have tried to do is to bridge the gap between individual and organisational reward by creating and rewarding a culture of continuous quality improvement with stretching goals and *measured outcomes* agreed in contracts with PCTs on an annual basis. Despite there being no individual reward through CQUIN to comply with increasing national guidance (including VTE risk assessment and treatment) for most healthcare professionals, different, less extrinsic motivational factors must influence uptake and compliance. These are discussed below:

1. In the case of Trust chief executives their pay and continuity in their post are often tied to the trust achieving certain targets. Since 2001, public sector hospitals have been given ‘star ratings’ according to their performance on targets and other indicators ^17^. These public service targets in health have commonly become known as "P45" targets - if you do not achieve set targets then your job may be at risk ^17^. The total reward package for Trust chief executives commonly includes basic pay, a spot rate salary determined by the role and an organisational weighting factor alongside an annual performance bonus scheme ^18^. CQUIN may be said to be one of those targets on which performance rewarded pay for chief executives is based. As a crude form of motivation it may be promulgated that if hospitals do not achieve their targets then the employment of the Trust chief executive may be at risk. Clearly this will have an impact on performance.

2. However, we may posit that for clinicians who do not receive any form of performance bonus like Trust chief executives, their motivational reasons for complying with NICE guidance may be more complex. Intrinsically, a concern for the welfare and treatment of patients has always been an over-riding factor in driving up standards in healthcare delivery. The NHS staff survey data for 2011 indicated that nine out of ten staff felt that their role made a difference to patients, with 69% saying they were able to deliver the quality of patient care to which they aspired ^15^. What is interesting from the perspective of this paper is the reduction in the number of staff in the same survey indicating their satisfaction with the level of freedom they now have to chose their own working methods (61%, down from 63% in 2010 ^15^). Here we may see the shift towards a greater standardisation of working practice against what were locally developed working practices for risk assessment and treatment of VTE ^19^.

3. As discussed earlier, CQUIN rewards are made available to providers through contracts with PCTs. Despite not rewarding the majority of individuals through personal rewards, the achievement of local quality improvement goals will increase overall hospital income. Because CQUIN funding is currently up to 1.5% of contract outturn value, conditional upon the achievement of quality outcomes specified in the contract ^20^, by achieving CQUIN goals healthcare professionals may be incentivised to adopt revised working practices in order to ‘earn’ extra monies at a departmental level in order to both improve their working practices and increase patient satisfaction. These may have taken the form of the employment of a lead specialist VTE nurse or improved staff and patient education.

4. A further motivating factor for the improvement of VTE risk assessment and treatment might be through the increased surveillance of clinical practice that the nationwide standardisation of VTE assessment now permits ^21^. Not only does this allow for ongoing intra-organisational surveillance through mandatory audit activity, but inter-organisational, public comparison of how well different hospitals risk assess and treat VTE also becomes possible. Measurements against national targets such as VTE risk assessment and treatment are now reported in annual hospital reviews of performance. As a result of increasing transparency in the outcomes of hospital performance, many healthcare professionals may have become increasingly aware of the need to drive up and maintain professional status by meeting local quality improvement goals in their hospitals.

This paper examines the organisational and workplace conditions that favour the implementation of national revised VTE guidance in 2010 in four hospitals in the greater South West of England. In particular, it focuses on the attitudes of staff towards changes to VTE risk assessment policies upon which CQUIN targets were based.

The implementation of evidence-based national guidance on VTE prevention across two specialities (general medicine and orthopaedics) were systematically compared in four hospitals in 2009 and re-assessed for 2010. The review used 816 randomly selected longitudinal case studies of the hospitals’ VTE prevention activities (in each of the four hospital sites 51 records for orthopaedics and 51 records for general medicine were audited for 2009 and 2010), complemented by a qualitative study into the practical responses by healthcare professionals to the revised national guidance and the imposition of payment by results (PbR). This paper suggests that in instances where there were unachievable clauses for achieving CQUIN rewards for VTE, there was less evidence of an improvement in VTE risk assessment and appropriate prescribing of venous thromboembolism prophylaxis.

## Research questions

Focussing on four hospital sites in NHS South West, our first research question was:

1. To what extent did the recording of VTE risk assessment and the prescribing of appropriate thrombo-prophylaxis increase after the imposition of revised NICE guidance in January 2010?

In so far as that did happen, we further ask:

2. In what respects was this increase a response to the financial incentives now available for acute providers through the CQUIN payment framework? Specifically:

a) What motivated staff to improve VTE risk assessment and prevention when reward was given at the level of the organisation rather than the individual?

b) Does potential public exposure of how well a hospital does in terms of meeting CQUIN targets for VTE risk assessment and prevention motivate staff to meet new NICE guidance, or:

c) Is concern for patient welfare at the forefront of staff motivation so any improvement in the recording of VTE risk assessment and prevention would have occurred even if incentivised payments were not in place?

## Methods

In order to compare the differences in the implementation of evidence-based national guidance on VTE prevention across general medicine and orthopaedics in four hospital study sites, a multi-method approach was adopted. To examine staff motivation in implementing (or not) the NICE guidance on VTE, qualitative methods were necessary. We content-analysed interviews and local documents expressing the attitudes, motives and rationales of the staff most responsible for implementing revised NICE guidance and meeting CQUIN targets. To examine how far staff in the study sites had in fact implemented the NICE guidance and responded to the CQUIN incentives, we extracted data from medical records.

### Sampling

As study sites, we sampled four hospitals with initially divergent models of VTE prevention activity. For example, Hospital A favoured universal prophylaxis with an ‘opt-out’ strategy where patients received prophylaxis unless contra-indicated. In contrast, Hospital C undertook early and ongoing risk assessment and only prescribed prophylaxis to patients with a clear, clinical need. Our sampling frame was a regional census undertaken in 2009 (reference not cited to protect anonymity of the sites) which reported organisational context and VTE prevention activity for each hospital.

It was decided to compare patients within general medicine and orthopaedics as it was hypothesised that a speciality with a relatively high proportion of planned admissions (orthopaedics) would find it easier to implement the revised guidance than a speciality with a relatively high proportion of unplanned admissions (general medicine). As we wanted to determine the extent to which the imposition of national guidance on VTE risk assessment and treatment had influenced the development and risk assessment intervention in each hospital we decided to sample records over two years. To minimise the effects of both the seasonal inflows of tourists and the rotation of junior doctors our sample was drawn from a list of all adult admissions for stays over 24 hours for the same three-week period in October 2009 and 2010.

We sampled staff using a snowballing method. We started by interviewing the medical and orthopaedics consultants leading VTE implementation and from their responses a care pathway for VTE risk assessment, prevention and treatment in each hospital site was able to be mapped. From this we were able to identify staff who made key decisions about VTE prevention and treatment during a hospital episode. These included: junior medical staff, VTE nurses, orthopaedic nurses, pre-operative assessment staff, pharmacists and senior hospital managers.

Each hospital approached our request to devise a sampling frame in different ways. Hospital A gave free access to admission data for the three week period in October from which we sampled the target population. Hospitals B, C and D would not release admission data but did provide a sample of patient files for audit. Subsequent statistical analysis confirmed that in those hospitals where we were unable to take our own sample, a random cross section of files appeared to have been made available without having been ‘cherry-picked’ by hospital records departments in order to show 100% compliance with national VTE risk assessment in orthopaedics and general medicine. An unexpected, later opportunity to compare our statistical analysis of VTE risk assessment in one of the hospital sites with an internal audit undertaken by some ward pharmacists during 2010 appeared to substantiate that the ‘cherry-picking’ of files to indicate stronger compliance was not the case.

To examine how the NICE and CQUIN norms were actually implemented we extracted from medical records the data shown on the data collection instrument (see data collection file). This was designed for mainly ‘tick-box’ entry of standard data entry fields. Power calculations indicated that a sample of 408 patients across all four hospitals for each year would be sufficient to detect an increase from 50% compliance in 2009 to 60% in 2010 with 80% power at the 5% level of significance. Data from 2009 allowed a retrospective sample predating NICE guidance 2010, whilst 2010 data was a prospective sample following NICE guidance implementation. In all 816 medical records were sampled.

Data extraction and interview schedule The first file shows the data extraction intrument used to collect data from medical records on the implementation of NICE and CQUIN norms. The second file shows the semi-structured interview schedule used to interview medical professionals about hospital compliance wit hthe 2010 NICE guidance.

### Data collection

Case study data were collected by interviews with key informants. We started collecting data in 2009 by interviewing either the medical or orthopaedic consultant responsible for leading VTE implementation in each hospital. A care pathway for VTE prevention and treatment was then mapped for both orthopaedics and general medicine in each hospital site. From this we were able to identify key healthcare professionals who appeared integral to VTE risk assessment and treatment throughout a hospital episode. We were then able to snowball to interview these professionals whose roles included: VTE nurses, pharmacists, orthopaedic nurses, pre-operative assessment staff and senior hospital managers. Forty four interviews were completed in the four hospitals over an 18 month period between March 2010 and July 2011. These were broken down hospital by hospital as follows: 11 each for Hospitals A and B; 10 for Hospital C and 12 for Hospital D. At certain times in Hospital A, time constraints in a busy Acute Assessment Unit meant that some interviews were undertaken with two members of staff at the same time. Fifty professionals were interviewed in total across the four hospitals. A semi-structured interview schedule was used (see interview schedule file). The interviews described the pre-NICE 2009 systems and reported how each hospital had responded to the 2010 NICE guidance and what factors appeared to facilitate or hinder compliance. All the interviews with key informants lasted approximately one hour and were conducted on a face-to-face basis in the four hospitals by SC or OB.

### Data analysis

Each interview was transcribed and checked for accuracy by the respondent. We used framework analysis by collating our interview data into tables for each hospital whose rows were categories that reflected our research questions. This approach enabled us to immediately ascertain whether there were any unforeseen emergent patterns in the data that necessitated us revising our initial assumptions. To check for accuracy, anonymised empirical findings were presented to the thrombosis committee in three out of the four sites – A, C and D.

### Hospital characteristics

A hospital can be awarded exemplar status as a ‘kite-mark’ of quality and good practice in VTE care. As part of the award they are expected to share examples of good practice, educational and audit material as well as providing advice on VTE care. A further expectation is that they collaborate on clinical research into VTE with other exemplar centres and develop innovate VTE prevention practices. In June 2010, there were sixteen VTE exemplar centres spanning the UK ^20^, including two of our hospitals (A and D).

Hospital A is a teaching hospital with over 900 beds. More than 48,000 patients pass through its doors every week. It provides a full range of acute and general hospital services for nearly half a million people. Speciality services are available to a greater population of over two million people. It employs approximately 5400 full-time staff. After becoming a Department of Health VTE exemplar site in 2009 it created a post of VTE Clinical Nurse Specialist to ensure it continued to comply with VTE risk assessment and disseminate best practice across the Trust.

Hospital B has over 750 beds spread between three geographically dispersed and semi-remote, rural sites (with the main site undertaking over 90% of the Trusts activity). It serves a population of 450,000 people (often doubled by holidaymakers at the busiest times of the year) and employs approximately 5200 staff. The recent opening of a new medical teaching facility had further enhanced its strong reputation for education and training. It was in the process of applying for Foundation Trust status at the time of this investigation.

Hospital C was one of the UK’s first foundation trust hospitals and provides acute hospital services to around 350,000 people as well as offering specialist services to a much larger population of approximately two million. Across all of its numerous sites it has over 30 wards, 20 operating theatres, 850 inpatient beds and 80 day-case beds. It employs approximately 6700 staff. There are over 310,000 consultant-led outpatient attendances per year and 120,000 day case admissions per year. It also has teaching hospital status.

Hospital D has foundation trust status and is much smaller in scale than the other three hospitals. It is not a teaching hospital and serves a population of approximately 225,000 spread across three neighbouring counties. Specialist services such as burns, plastic surgery, genetics and rehabilitation extend to more than three million people within a greater geographical area. It employs around 3900 full and part-time staff and had been awarded Department of Health exemplar status for VTE in 2009 (like Hospital A). It continued to be at the forefront nationally on VTE prevention and hosted a national VTE conference in 2010.

## Ethical considerations

Ethical approval was not needed for this study as the local NHS Research and Development Service deemed it to be service evaluation and audit. Honorary contracts for the two researchers (SC and OB) were necessary in three of the four hospitals (B, C and D). The fourth hospital (A) only required a letter of access.

All four hospitals were assured that both they and individual informants would not be identifiable in both data analysis and publications. Researcher safety was addressed by ensuring that someone within the team had knowledge of the date, time and place of interviews.

## Findings

1. Increase in the recording of VTE risk assessment and prescribing of appropriate thrombo-prophylaxis.

All four sites show an improvement in the percentage of patients risk assessed for VTE between 2009 and 2010. Table 1 shows the overall pattern of convergence with NICE requirements for VTE risk assessment and the amount of CQUIN monies available for each hospital if local quality improvement goals were met. It also indicates the percentage of CQUIN monies allocated to further improving VTE risk assessment by paying for a Lead VTE Nurse or improving staff education in VTE.

**Table 1.** Risk assessment changes in all hospitals between 2009 and 2010 and CQUIN targets for each.

Site	All	Hospital A	Hospital B	Hospital C	Hospital D
**2009 - % of patients risk assessed for VTE**	51.5	43.1	58.8	32.4	71.6
**2010 - % of patients risk assessed for VTE**	79.2	90.2	83.3	68.6	74.5
**Significance(p<0.05)**	+	+	+	+	0
**VTE Clinical Nurse Specialist in Post?**	n/a	Yes	No	No	No
**CQUIN target payments in £000s**	n/a	500	400	300	Not eligible
**CQUIN target achieved?**	n/a	Yes	Yes	Yes	n/a
**Any CQUIN monies used to improve VTE risk assessment?**	n/a	No	No	No	n/a

The biggest overall percentage change in risk assessment was in Hospital A. This was not unexpected. Despite its low figure of 43.1% for risk assessment in 2009, it had been awarded Department of Health VTE exemplar site status in December 2008 due to its policy of prescribing universal pharmaceutical prophylaxis unless contra-indicated. However, poor documentation of risk assessment in patient files meant that we were unable to count that a VTE risk assessment had taken place for many patients for our audit purposes in 2009. This might begin to explain the low 43.1% statistic. Over the 12 months of our audit, it rose from last to first place for VTE risk assessment in the four hospitals in our study as auditable risk assessment rose to 90.2% in 2010. In contrast, Hospital D, also a Department of Health exemplar site for VTE pre-2010 NICE guidance was the leading hospital in 2009 by risk assessing 71.6% of inpatients for VTE. Only a small improvement was able to be statistically shown for 2010 as auditable risk assessments rose to 74.5%. Again, we were only able to count patient files where there was written evidence of an actual risk assessment being undertaken. This statistically non-significant shift in risk assessment might be explained by inaccurate recording of paperwork or poor filing of risk assessment documentation. However, Hospital D was the only hospital to not be eligible for CQUIN incentives and this may have affected the motivation for implementing the required national changes in completing VTE risk assessment documentation.

2. How far was the improvement in VTE risk assessment a response to the financial incentives paid to acute providers through CQUIN?

All hospitals had local VTE prevention policies in place before national guidance was introduced in 2010. Three of the hospitals (B, C and D) had developed policies that ultimately matched the national standards subsequently set by NICE in 2010. These policies were built on slightly differentiated risk assessments that were often individual for each specialism. For example in Hospital D, Obstetrics and Gynaecology used a VTE risk assessment they had developed from the Royal College of Obstetricians and Gynaecologists risk assessment tool rolled out in 2009 ^22^. When questioned about the use of differing risk assessment forms across the hospital during interview, the chair of the thrombosis committee at Hospital D described these differentiated risk assessments thus:

"… *(the) end output of the algorithm may vary by speciality but not the input.*"

This suggests that risk factors across specialisms might vary, but the overarching aim to prevent VTE during a hospital episode remained consistent throughout the hospital. Prevention of venous thromboembolism was therefore based on the cumulative risk factors of assessment and treatment.

In contrast to Hospital D, Hospital A had developed an ‘opt-out’ policy where *all* patients (irrespective of admittance speciality) were given a standardised dose of pharmaceutical prophylaxis unless it has been contra-indicated. This ‘opt-out’ policy had originally been devised by the thrombosis committee following a national campaign around the preventable risks of VTE. However, such a blanket policy meant individual risk assessment for VTE was not always officially recorded in hospital notes nor, therefore, reflected in our compliance scores (as shown in Table 1) for 2009 (which appeared to be low in comparison to Hospitals B, C and D). As new drug charts were introduced in Hospital A during 2010 which included VTE risk assessment proformas that needed to be completed before any drugs were prescribed, our audit was able to document a large increase in evidence of risk assessment from 43.1% in 2009 to 90.2% in 2010.

Our analysis appears to support the suggestion that the imposition of national guidelines for VTE risk assessment in 2010 acted as the driving force behind the significant improvements in the recording of risk assessment in Hospital A between 2009 and 2010 as outlined above. The recording of risk and prescription of appropriate VTE prophylaxis appeared to be further underpinned across all the hospitals by the introduction of the CQUIN goal on VTE risk assessment.

Hospital A achieved all of its CQUIN targets for VTE risk assessment, resulting in the reward of £500,000 to the hospital operating budget. A member of its VTE committee suggested that as the hospital had performed so well with meeting current CQUIN standards, the PCT would be very unlikely to include as many targeted criteria in following years. The hospital would probably achieve them all thereby making financial incentives too expensive to finance. When asked in early 2012 if any of the £500,000 CQUIN monies had been directly received by the thrombosis committee to improve VTE risk assessment or treatment the committee member replied:

" *The VTE prevention initiative has received nothing from the Trust so far but talks are in progress*."

Hospital B also received payment for achieving CQUIN targets for VTE. The 2010–2011 VTE figure was approximately £400,000. However, as was the case in Hospital A, the Chief Haematologist at Hospital B said:

"… *not a penny has come to our department [haematology] as I have been told there is no linkage between the monies and its use in the specified area*."

In Hospital C the value of the Trust’s VTE CQUIN reward for VTE was in the region of £300,000. In line with our findings in Hospitals A and B, the VTE CQUIN monies earned to date in Hospital C had not been used to explicitly invest in VTE assessment. However, an electronic system for capturing VTE risk assessment was in the course of development but the hospital was unable to confirm if research and development monies for this initiative had come directly from CQUIN payments.

However, what was very interesting from the point of view of our research was that Hospital D was found to differ significantly from the previous hospitals discussed above as they were said to have experienced a ‘difficult relationship’ with their PCT over a number of years and:

"… *therefore one of the consequences of that is an unachievable clause for achieving VTE CQUIN money has been set into the contract which is outside our control and that is ambulance response times on patients who require thrombolysis*." (Lead Clinician for Haematology, Hospital D).

This resulted in no CQUIN monies being made available to the hospital whether they achieved CQUIN targets or not.

Despite the improvements in VTE risk assessment and prevention discussed above, it remains difficult to ascertain with certainty if monetary incentives *were* the driving force behind the significant changes in the percentages of patients risk assessed between 2009 and 2010 in each hospital. We used interview analysis to try and determine if a more intrinsic type of motivation in driving up patient safety might explain the improvements in risk assessment. A Lead Anti-Coagulant Nurse from Hospital B suggested that CQUIN incentives were actually a crude form of payment by results. His concern appeared to focus on whether any monies obtained by CQUIN would be used to improve VTE risk assessment by employing more staff (as evidenced by Hospital A using the funding to employ a Lead VTE Nurse) or simply swallowed into general operating budgets:

" *I think in the current financial climate you need to be realistic that money will be absorbed but at the same time… it’s a fairly significant amount and you would not need every penny of that money to deliver an effective VTE role. I think what you need is the hospital to be able to say we are prepared to spend what is needed to achieve it and whatever is left over we will absorb that as a hospital. That’s acceptable.*"

Certainly in the three hospitals eligible for VTE CQUIN funding all monies had indeed been swallowed into the general operating budget so it was difficult to ascertain whether extrinsic motivation in the form of payment by results drove increased compliance to NICE guidance.

We suggest that hospitals were not using CQUIN rewards to improve the patient experience by allocating CQUIN payments to specific departments. Indeed interview data from a Pre-Operative Assessment Nurse in Hospital B suggest that even when CQUIN targets were met the amount of time being allowed for VTE risk assessment in all sites had since been *reduced* to allow for a greater volume of patient activity, whilst at the same time staff were being expected to *increase* their recording and frequency of VTE risk assessment in order to comply with national guidelines on which CQUIN rewards were based:

*"…our processes have changed quite considerably in the past three or four years to actually increase the throughput of patients that we see… historically… the doctors that were in clinic would always do a risk assessment [for VTE] so you would always see the bottom of the prescription chart you know – high risk – and then we would measure for the TED stockings but we don’t now*." (Sister at Pre-Operative Assessment Clinic, Hospital B).

This finding appears to fit with the NHS Staff Survey (2010), which suggests that just under half of all staff (46%, up from 45% in 2010) felt they do not have enough time to carry out their work and 42% indicated they could not meet all the conflicting demands on their time at work. Yet, paradoxically, 87% of staff indicated they were satisfied with the quality of care provided to patients ^15^.

## Conclusions

The research–practice gap is widely documented. It can be extremely difficult to implement even those strategies (such as risk assessing for VTE) that are known to carry large health benefits into the healthcare system. The general goal of VTE risk assessment for all patients appears to have been transformed into a metric-specific aim statement with the process measure defined as 90% risk assessment of all in-patients. Despite this, recent figures from the Department of Health show that since national measures were introduced in 2010, patients are now twice as likely to be risk assessed for VTE. More than 14.3 million patients have been risk assessed since the measure was put in place, and an estimated 230,000 patients are being screened per week. So our findings appear to match events in the NHS more widely.

As our data show, the introduction of CQUIN targets has focussed the attention of hospital management towards improving the recording of risk assessment and the prescribing of appropriate VTE prophylaxis. The role of CQUIN has been re-emphasised as being a key mechanism for delivering the improvements identified as markers of quality by NICE quality standards. However, there appears to remain a contradiction between the more traditional, extrinsically motivated payment by results, to reduce waiting times and increase the volume of work completed in a given period, and the development of a more intrinsically motivated effective patient experience by driving up standards. The improvements in VTE risk assessment between 2009 and 2010 in each hospital suggests that the implementation of national clinical guidelines for VTE risk assessment and appropriate prescribing of prophylaxis appears to have significantly changed the attitudes and behaviour of healthcare professionals towards VTE risk assessment. In turn, this has increased compliance with national guidelines. However, it is very difficult to separate whether improvements in VTE risk assessment and the prescribing of appropriate thrombo-prophylaxis has increased due to risk assessment for VTE becoming a national performance indicator underpinned by the introduction of a national CQUIN goal on VTE risk assessment (motivated by the potential of extrinsic financial reward) *or* through the continuous improvement in the risk management of VTE in each hospital (intrinsic motivation). The increased measurement and risk assessment and the increased transparency of clinical practice appear to have created what proponents of Foucault (a 20 ^th^ century philosopher who focussed on the methods by which power is conveyed in society), call a ‘disciplinary’ motive for compliance with NICE and CQUIN norms ^23^. Such motives are certainly intrinsic but not in the positive way that the original theorists of intrinsic motivation seem to have had in mind.

Certainly increased levels of risk assessment and prophylaxis for VTE are evident from our study, but once again, it is difficult to establish with certainty whether clinical activity has actually changed, or simply the recording of it. Whether a year is long enough for the full impact of guideline implementation to be apparent is difficult to say, although our audit of medical records does show substantial changes in the numbers of patients being risk assessed and appropriately treated. Our study appears to mirror that of Tharvarajah and Wetherill who found that by attaching a VTE risk assessment tool to drug charts significant improvements in the recording of risk assessment can be observed ^24^.

What would be interesting for future investigation would be whether the focus of hospital management to comply with national policy guidance on VTE risk assessment and prophylaxis remains after the financial ‘carrot’ of CQUIN monies has been withdrawn. To what extent are we able to determine whether the culture of VTE risk assessment will have become so entrenched in the working practices of healthcare professionals that we will continue to see compliance with *quality* guidelines that may no longer be rewarded financially?
