# Supplementary material for: Has incentive payment improved venous thrombo-embolism risk assessment and treatment of hospital in-patients?
Source: F1000Res. 2013 Feb 12;2:41. [Version 1] doi: 10.12688/f1000research.2-41.v1 (PMC3790600; doi:10.12688/f1000research.2-41.v1)
Supplement: Data extraction and interview schedule — The first file shows the data extraction intrument used to collect data from medical records on the implementation of NICE and CQUIN norms. The second file shows the semi-structured interview schedule used to interview medical professionals about hospital compliance wit hthe 2010 NICE guidance. [file f1000research-2-308-s0000.tgz › Data_extraction_instrument.pdf]

Data extraction instrument

Inpatient Medical Record Data Extraction Instrument

Demographics

1. Case identifier [……….]
2. Age / sex [. . .] [M/[F]
3. Referral Source [GP: A&E: ITC: Other (say which)]
4. Admission date [--/--/--]
5. Specialty [general medical / orthopaedics/ other (say which)]
6. Consultant:
7. Main diagnosis (ICD10 code) [….]
8. Main procedure (procedure code) [….]
9. Discharge date [--/--/--]

VTE Prophylaxis Implementation

10. DVT risk assessed (tick all that apply):
    (a) At outpatients appointment (not specifically for DVT)? [Y/N/No Info]
    (b) At DVT clinic? [Y/N/No Info]
    (c) On initial admission to ward (medical / surgical take)? [Y/N/No Info]
    (d) After transfer to another ward? [Y/N/No Info]
    (e) Immediately before discharge? [Y/N/No Info]
    (f) Other time(s) (say which)? [Y/N/No Info]

11. Decision on prophylaxis:

Did Risk Assessment indicate patient needed VTE prophylaxis? [Y/N/Not recorded]

(Take 'major risk' and 'medium risk' (or equivalent terms) as indicating need for prophylaxis. Take 'minor risk' (or equivalent term) as meaning no need for prophylaxis.)

How long were pharmaceuticals for VTE prophylaxis prescribed / given for?

(a) Did clinician decide prophylaxis was contra-indicated? [Y//Not Known]

If 'YES' GO TO q13

(b) If prophylaxis was indicated but the *[locally adopted protocol]* was not followed, was a reason for non-compliance also recorded?

[Y/N]

IF 'YES' GO TO q13

12. Which of the prophylaxes stated in the *[locally adopted]* guidelines was given (tick all that apply)? If no information provided assume no prophylaxes given.

(a) Patient mobilised within 24 hours? [Y/N]
(b) Low molecular weight heparin prescribed? [Y/N]
(c) Aspirin prescribed? [Y/N]
(d) Warfarin prescribed? [Y/N]
(e) Anti-embolism stocking(s)? [Y/N]
(f) Venous foot pump(s) or Intermittent pneumatic compression? [Y/N]
(g) Oral anticoagulant bridging? [Y/N]
(h) Inferior vena cava bridging? [Y/N]
(i) Other (say what)? [Y/N]

13. Did discharge letter/discharge summary/electronic summary advise GP on VTE risk? [Y/N]

14. Did discharge letter/discharge summary/electronic summary advise GP on VTE prescribing? [Y/N]

If yes, for how long was it prescribed?

15. Any other note / comment [e.g. oral v subcut etc.]:

Compliance score (maximum 4 points):

1 point if at least one item ticked 'yes' under (10) above.

1 point if one item ticked under 'yes' under (11) above.

1 point if at least one item ticked under (12) above.

1 point if 'Y' at q13.

1 point if 'Y' at q14.
