# Supplementary material for: Has incentive payment improved venous thrombo-embolism risk assessment and treatment of hospital in-patients?
Source: F1000Res. 2013 Feb 12;2:41. [Version 1] doi: 10.12688/f1000research.2-41.v1 (PMC3790600; doi:10.12688/f1000research.2-41.v1)
Supplement: Data extraction and interview schedule — The first file shows the data extraction intrument used to collect data from medical records on the implementation of NICE and CQUIN norms. The second file shows the semi-structured interview schedule used to interview medical professionals about hospital compliance wit hthe 2010 NICE guidance. [file f1000research-2-308-s0000.tgz › Interview_schedule.pdf]

VTE protocol compliance: organisational data interview schedule

Checklist of topics *Interviewer to select* ad hoc *which of these to pursue with particular informants, according to what appears relevant to the informant's role, the nature of the particular study organisation and what data are already available to the researchers.*

[Background]

- Of the patients you treat, approximately what proportion would you estimate to be at risk of VTE?
- How many of your patients develop VTE as in-patient or out-patient?
- What do you regard as the main adverse consequences of this risk of VTE?
- What are the other main risks that your patients face during their time in hospital?
- In your view, what priority should be given to preventing VTE compared with avoiding these other main risks?
- To what extent have 'difficult choices' to be made, in terms of having to decide between avoiding VTE and avoiding some other risks (e.g. post-operative bleeding)?
- What might be the adverse of VTE prophylaxis? Have you witnessed any of these?

[Risk Assessment]

- By what method(s) do you assess patients' risk of VTE?
- Do you use a standard risk assessment tool? If so, which one? Where do you obtain blank copies (e.g. included in admission documents)?
- Who actually undertakes the risk assessment? (Consultants? Other medical staff? Nurses? Somebody else?)
- At what stage(s) in the patient episode does that happen?
- How is the patient's risk recorded?
- Under what circumstances is the risk of VTE discussed with the patient?
- Does [*hospital name*] monitor the extent to which you undertake the VTE risk assessment?
- When (or is) the risk assessment repeated?

[Prophylaxis]

- What VTE prophylactic measures do you generally use?
- Are there any methods of VTE prophylaxis that you generally avoid using? Why?
- Are there any protocols or guidelines for VTE prophylaxis that you normally follow? Why these?
- Would you wish to see any changes to that protocol? Why?
- What is the application rate for VTE prophylaxis in your department? How would you improve this?

[Discharge]

- Are patients informed of the symptoms of post-discharge complications? By whom? And by what method(s)?
- What do you tell patients who you have identified at being 'at risk'?
- Do you inform GPs about what prophylaxis given or when extended prophylaxis is needed?

[Management]

- Have the arrangements changed over the last year? If they have changed, why was that?
- What training on VTE risk prevention or guidelines did you have?
- What organisational arrangements or (lack of) resources hinder you in following VTE protocol?
- If you could change one thing in the way this hospital goes about preventing VTE, what would it be?
- *Are there any other important aspects of VTE prevention that you would also like to mention?*
