# Supplementary material for: Has incentive payment improved venous thrombo-embolism risk assessment and treatment of hospital in-patients?
Source: F1000Res. 2013 Feb 12;2:41. [Version 1] doi: 10.12688/f1000research.2-41.v1 (PMC3790600; doi:10.12688/f1000research.2-41.v1)
Supplement: Peer review history

# Peer review: Referee response for version 1

**Published:** 2013-02-28

**Authors:** Lynda Bonner

**Affiliations:** 1 King's Thrombosis Centre, Kings College Hospital NHS Foundation Trust, London, UK

This is an interesting mixed methods study. It provides a comprehensive background to incentive payments (payment by volume and payment by results) in the NHS. The authors mention that compliance with VTE CQUIN targets 2010-11 was measured through a monthly audit of patients who received appropriate thrombo-prophylaxis after risk assessment, however, it is worth adding that the financial incentive for reaching a 90% threshold for VTE risk assessment was based on **census** data collection and that Unify2 contains data on the VTE risk assessment indicator only. 

The section on motivation in the NHS is particularly relevant to those who are in a position to use CQUIN as a driver to lead the implementation of effective VTE prevention practice in their organisation. The power of CQUIN relies on its meaningful translation of its implications for staff within the healthcare organisation. The authors give examples of how CQUIN can act as a motivator to engage different staff groups that have a role in VTE prevention and suggest that there was less evidence of improvement where unachievable clauses for achieving CQUIN rewards existed. This highlights the importance of commissioners and providers agreeing realistic targets to activate the motivational potential of CQUIN. 

The study methodology appears justified and sound. Any limitations in methodology were acknowledged and where possible action was taken to minimise their potential impact. Ethical issues were considered and authorisation for the study was granted by the appropriate authorities. Findings are discussed within the wider context of practice nationally and debate created around the intrinsic/extrinsic motivations that may have been triggered by payment by results. 

It might be worth reflecting on the authors positive view of CQUIN as a financial ' **carrot**' and whether this may have influenced their interpretation of findings related to motivation. (This is in contrast to the more negative view held by some that CQUIN is a financial ' **stick**' whereby monies allocated for an organisation will be withheld if they are unable to meet their targets). The snowball sampling technique could have limited the richness of data that might have been gathered from untapped sources. 

This study adds to the current knowledge base and its findings support a positive correlation between a statistically significant improvement in the percentage of patients risk assessed for VTE and the achievement of CQUIN payments amongst the study sample. It also offers an insight into the perceptions of CQUIN as a motivator and acknowledges that further research in the area is needed. It should be of genuine interest to those in the field of VTE prevention or to those who are aiming to achieve CQUIN payments for their organisation.

I have read this submission. I believe that I have an appropriate level of expertise to confirm that it is of an acceptable scientific standard.

# Peer review: Referee response for version 1

**Published:** 2013-02-19

**Authors:** Oweikumo Eradiri

**Affiliations:** 1 Colchester General Hospital, Colchester, UK

- Summary – The article is a robust presentation of current challenges in implementing strategies for effective VTE prophylaxis. I consider it an important contribution to the debate on incentivising managers of NHS Trusts to support clinicians in their bid to innovate for patient safety, or comply with national quality improvement guidance.  
- Methodology – I have not reviewed the power calculations, but the overall approach is sound.
- Recommendation – The article could perhaps have benefited from additional answers to research questions 2b and 2c.

I have read this submission. I believe that I have an appropriate level of expertise to confirm that it is of an acceptable scientific standard.
